# Prevalence and Risk Factors for IgE and IgG Cow's Milk Protein Allergies in Saudi Arabia

**DOI:** 10.7759/cureus.50812

**Published:** 2023-12-19

**Authors:** Mashail A Baghlaf, Noura M Eid, Sumia Enani, Samia Kokandi, Bakr Alhussaini, Mashhoor S Waked

**Affiliations:** 1 Department of Medical Laboratory Sciences, Faculty of Applied Medical Sciences, King Abdulaziz University, Jeddah, SAU; 2 Department of Clinical Nutrition, Faculty of Applied Medical Sciences, King Abdulaziz University, Jeddah, SAU; 3 King Fahd Center for Medical Research, King Abdulaziz University, Jeddah, SAU; 4 Department of Food and Nutrition, Faculty of Human Sciences and Design, King Abdulaziz University, Jeddah, SAU; 5 Food, Nutrition, and Lifestyle Unit, King Fahd Center for Medical Research, King Abdulaziz University, Jeddah, SAU; 6 Department of Nutrition, International Medical Center, Jeddah, SAU; 7 Department of Pediatric Gastroenterology, Faculty of Medicine, King Abdulaziz University, Jeddah, SAU; 8 Laluna Clinic, Healthcare Center, Jeddah, SAU

**Keywords:** nutrition, allergy, adults, pediatrics, cow’s milk protein allergies

## Abstract

Cow's milk protein allergies (CMPAs) particularly occur in infancy and early childhood due to an immunological allergic reaction to milk proteins. This issue is increasing in Saudi Arabia and requires research to improve health status and provide alternatives. Our study aims to investigate the important aspects of immunoglobulin E (IgE) and IgG CMPA in Saudi Arabia regarding its prevalence and association with demographic and health-related factors in both adult and pediatric populations. A descriptive retrospective cross-sectional study was conducted on 376 patients, comprising 314 adults aged between 19 and 86 years, and 62 pediatric patients aged between one and 12 years. The study focused on individuals who attended a private medical center in Jeddah city over the past five years. Laboratory results for food allergy of IgE and IgG tests, including cow's milk proteins (CMPs), serum 25-hydroxyvitamin D (25OHD), specific IgE inhalant allergy results, and other health-related factors were collected from an electronic record system. Results have shown that casein was the most common sensitizing allergen of CMPs in adults, whereas α-lactalbumin was the most common sensitizing allergen in pediatrics. The most frequent sensitizing allergen in IgG CMP was cow's milk in 54/92 (58.7%) adults, followed by cow's sour milk products 41 (44.6%). Cow's milk was the most common sensitizing allergen in 20/20 (100%) children. The rate of CMPA was significantly higher in children younger than five years old (*P* =0.003), while children who interacted with pets had a marginal significantly reduced rate (*P* = 0.054). Thus, cow's milk is the most sensitizing allergen in IgG CMPs in adults and pediatrics.

## Introduction

Food allergies are on the rise and have become a major public health issue that has attracted the attention of many health professionals and researchers in the fields of medical sciences and nutrition [1]. This trend has been observed in both Westernized and other developed countries [2], where there are guidelines and policies in place. The Saudi Food and Drug Administration (SFDA) imposes regulations to protect people with allergies, such as ensuring appropriate and clear labeling of allergenic ingredients in food products and the raw materials used in these products [3]. However, several types of allergies, or so-called *sensitivities*, have been detected recently among infants, children, and adults [1], including cow's milk protein allergies (CMPAs) [2]. In identifying the reasons behind this persistent rise in allergies, immunological, genetic, and risk factors, as well as other related health and dietary issues, need to be considered. 

CMPA can be provoked by different immunological pathways. The immunoglobulin E (IgE)-mediated type is elicited by IgE and is responsible for about 60% of cases. This type of allergy is characterized by manifestations that appear within two hours of exposure to allergenic proteins. In the other type of CMPA (the non-IgE-mediated reaction type), manifestations could occur between three hours and one-week post-ingestion of the allergenic proteins, mostly involving gastrointestinal symptoms [4,5]. The reactions involve several immunological mechanisms such as antibody-mediated responses, including type II and type III hypersensitivity reactions that are mainly mediated by IgM and IgG; in addition to other mechanisms such as the production of IgA [1,4,6]. IgG-mediated food allergies occur when increased gut permeability enables particles of food allergens to gain access to the blood circulation, thereby stimulating the development of IgG [7-9]. However, the mechanism of IgE-mediated reaction has been well discussed in many studies, whereas the IgG-mediated type is still not fully understood, though mentioned in a few studies that identified specific IgG subclasses, namely, IgG1 and IgG4, which produce reactions to α-lactalbumin and β-lactoglobulin milk protein [10-12].

To prevent such types of allergies, an elimination diet is suggested by specialist organizations and guidelines. This prescribes the total avoidance of cow’s milk from the patient’s or child’s and mother’s diet, along with the use of appropriate cow’s milk substitute formulas such as extensive hydrolyzed formula (EHF) and amino acid formula (AAF) [13,14]. Guidelines also recommend breastfeeding an infant solely for four to six months and delaying the introduction of exogenous proteins and solid foods [9,15,16]. Recent studies showed that the addition of probiotics and prebiotics to a patient’s diet helps to prevent food allergies. However, supporting studies are few, as the World Allergy Organization (WAO) attests [9,17,18].

This study aims to provide insights into the prevalence of both IgE and IgG types of CMPA in Saudi Arabia as well as the relationship between CMPA and gender, body weight status, vitamin D level, and environmental risk factors in both adult and pediatric populations.

## Materials and methods

Participants

Participants were gathered from an electronic record system for a descriptive retrospective cross-sectional study conducted in the year 2020. The study involved a total of 376 patients, comprising 314 adults and 62 pediatric individuals, who sought care at Laluna Clinic, a private medical center specializing in allergic diseases in Jeddah, located in the western region of Saudi Arabia. Data were collected retrospectively from 2020 to 2023. These data were extracted from the electronic record system. We collected data for adult patients aged 19 to 86 years and pediatric patients aged nine months to 12 years who had laboratory test results for food allergy of IgE or IgG and compared it against common food allergy triggers, including cow's milk proteins (CMPs) panel. Other laboratory parameters were also collected, including the serum 25-hydroxyvitamin D (25OHD), total IgE, total IgG, and specific IgE inhalant allergy test results. In addition, patient demographics, body mass index (BMI) calculations, and reported clinical symptoms were also collected for each patient. Patients with missing results for both food sIgE and food sIgG laboratory tests, as well as those with repeated medical files, were excluded. 

The diagnosis of CMPA was based on the existence of positive results of CMP-specific IgE or IgG test or both. Samples were deemed positive when serum titer concentration of IgE or IgG immunoglobulins against one or more CMPs was detected and exceeded the reference cut-off titer provided by the manufacturer.

The sample size calculation was adapted from previous studies with a total of 100 pediatric groups [19] and 100 adult groups [20] required to detect a prevalence with an alpha of 0.05 (two-sided) and a power of 0.80.

Determination of specific IgE and IgG (allergy testing)

sIgE against both food and inhalant panels was tested using 300 μL of patients' serum samples that were analyzed using the AlleisaScreen® system from Mediwiss (Analytic, Moers, Germany). This test is an enzyme immunoassay on a nitrocellulose membrane (immunoblot) for in vitro quantitative detection of allergen-specific IgE antibodies in human serum. In a reaction trough, allergens were attached to the surface of nitrocellulose membranes. The patient's serum was pipetted into the reaction trough and incubated to allow allergen-specific IgE antibodies to bind to the nitrocellulose membranes via the allergen. Nonbound material was removed by washing. After this, a biotin-coated anti-human IgE antibody was added. Washing removes nonbound detector antibodies. Next, streptavidin-conjugate was added, and nonbound streptavidin conjugate was removed by washing. This step was followed by the addition of a developing agent, resulting in the production of a particular enzymatic color reaction of the alkaline phosphatase. The coloring was proportional to the amount of particular antibodies in the serum sample. Improvio Scanner, which takes a snapshot of the membrane, was used to evaluate the test strip after it completely dried. The system was available with different and customizable sets of food allergen panels with up to 60 separate allergens, including CMPs, fruits, vegetables, and seafood. CMP panels in this system included milk, casein, α-lactalbumin, and β-lactoglobulin. The strip AlleisaScreen panel contained the following aeroallergens: house dust mites and cockroach panel (Dermatophagoides pteronyssinus, Dermatophagoides farinae, and cockroach), animal dander panel (cat, dog, horse, and camel's hair), tree and grass pollens panel (oak, olive, Bermuda grass, and ryegrass), and molds panel (Penicillium notatum, Aspergillus fumigatus, Cladosporium herbarium, Alternaria alternata).

The AlleisaScreen detection system offers advantages such as a convenient procedure and reliable results. The AlleisaScreen system assay was calibrated against the international reference preparation *2nd WHO IRP 75/502 for specific IgE*. Depending on AlleisaScreen recommendation, the values were expressed as IU/mL, and results under 0.35 IU/mL were considered negative and scored as class 0, and those between 0.35 and 0.69 IU/mL scored as positive results with severity class 1. Values between 0.70 and 3.49 IU/mL scored as class 2, values 3.50 and 17.49 IU/mL as class 3, and values 17.50 and 49.99 IU/mL as class 4. Values 50.00 and 99.99 IU/mL as class 5, and values above 100 IU/mL were considered extremely positive and categorized as class 6. 

The sIgG against common food triggers in this study was assessed by the ImuPro food allergy test by R-Biopharm AG, Germany. The test was based on enzyme-linked immunosorbent assay (ELISA) to measure IgG antibodies to the food allergen in patients' blood. The sample contained specific food IgG to certain food bound to the food allergen found in the test microplate forming a complex, which was then measured by colorimetric assay. This detection system provided a wide range of food panels with up to 270 food allergens, including CMP products such as cow's milk, Rennet cow cheese, and sour cow milk products. Based on the titration of IgG antibodies, results were categorized into three levels: not elevated IgG < 7.5 μg/mL, elevated IgG ≥ 7.5 μg/mL, and highly elevated IgG ≥ 20 μg/mL.

 

Measurement of total serum IgE and total serum IgG

Total IgE and total IgG were assessed for 224 patients using the Cobas e601 device (Roche Diagnostics GmbH, Germany). The device utilizes electrochemiluminescence technology for immunoassay analysis using serum/plasma samples for the qualitative and quantitative determination of immunoglobulins. According to the manufacturer's recommendation, a total IgE level below 100 was considered normal for the adult population. For pediatric patients, the reference range was as follows: one to five years, up to 60; six to nine years, up to 90; and children aged from 10 to 15 years, up to 200. 

Determination of Serum 25OHD

25OHD was measured for 120 patients using serum/plasma samples and analyzed by LIAISON® 25 OH Vitamin D Total Assay from DiaSorin, Saluggia, Italy. This analysis used chemiluminescent immunoassay technology for the quantitative determination of 25OHD. The concentration of 25OHD was expressed in ng/mL. The following ranges for 25OHD status categorization were recommended based on literature reviews: deficiency when 25OHD was less than 10 ng/mL; insufficiency when 25OHD was between 10 and 30 ng/mL; and sufficiency when 25OHD was between 30 and 100 ng/mL [21]. 

Type of infant delivery and pattern of child’s feeding survey

A telephone survey was conducted to collect information about the type of delivery of the participant’s child and the feeding patterns the children received during their first year of life. In addition, the survey explored the presence of a family history of allergies in parents or siblings and investigated the presence of pets in the affected child's home.

Statistical analysis

Descriptive statistics summarized participants' characteristics using mean (standard deviation [SD]) or median (interquartile range [IQR]) as appropriate. Pearson’s chi-square test was conducted to identify the possible association between the presence of CMPA allergies in both forms IgE and IgG with gender, BMI, and vitamin D in the adult population. Type of child delivery, the pattern of feeding, the presence of a pet in the affected child's home, and the presence of a positive family history of allergy in the parents or siblings were also investigated for possible association among the pediatric population using Pearson’s chi-square test. Fisher's exact test was used when needed. A *P*-value of <0.05 was considered significantly different. All statistical analyses were carried out using IBM SPSS Statistics for Windows, Version 26 (IBM Corp., Armonk, NY). All graphs were created using IBM SPSS Statistical Software and Excel Statistical Analysis.

## Results

Adult population

Descriptive Statistics

Three hundred and fourteen adults (female 186, 59.2%; males 128, 40.8%) were included in this study. Table 1 summarizes the age, gender, weight, and height of adult participants included in this study. BMI was only reported for 98 (31%) participants. The majority of the participants (70/98, 71%) were either overweight or obese.

**Table 1:** Demographic characteristics of adult participants enrolled in the study. SD, standard deviation; BMI, body mass index

Demographic characteristics	Mean ± SD
Age (years)	39.9 ± 13.2
Male, *n* (%)	128 (40.8 )
Female, *n* (%)	186 (59.2)
Weight (kg)	74.4 ± 19.3
Height (cm)	162.1 ± 10.1
BMI (kg/m^2^)	28 ± 6
Underweight, *n* (%)	6 (6.1)
Normal, *n* (%)	22 (22.4)
Overweight, *n* (%)	35 (35.7)
Obese, *n* (%)	35 (35.7)

Prevalence of CMPA in Both IgE and IgG Forms

Prevalence of IgE CMPA: Of the 314 adults enrolled in this study, the food sIgE level was measured in 295 (94%) of them. Table 2 summarizes the prevalence of CMPA sIgE in adults for milk, α-lactalbumin, β-lactoglobulin, and casein allergens. Casein was the most common sensitizing allergen (43, 14.6%) followed by α-lactalbumin (40, 13.5%) and milk (22, 7.5%). More allergy cases were significantly reported against all food allergens present in the sIgE panel, including CMP profile (casein, α-lactalbumin, milk, and β-lactoglobulin) in the adult population. These findings were statistically calculated using a chi-square goodness-of-fit test followed by post hoc examination using partitioning with the Bonferroni-adjusted *P*-value (0.05/29). Table 3 shows the prevalence of CMP-sensitizing allergens in adults among other food allergens present in the sIgE blood test panel.

**Table 2:** The prevalence of sIgE CMPA in adults for milk, α-L, β-L, and CA allergens (n = 295). ^*^Missing values are not considered in calculating the percentages. α-L, alpha-lactalbumin; β-L, beta-lactoglobulin; CA, casein; CMPA, cow's milk protein allergy; CMP, cow's milk protein

Cow's milk proteins (CMPs)	IgE CMPA reported cases, *n* (%)
Milk	22 (7.5%)
α-L	40 (13.5%)
β-L	20 (6.8%)
Casein	43 (14.6%)
Milk + α-L	13 (4.4%)
Milk + β-L	3 (1.0%)
α-L + β-L	7 (2.4%)
Milk + CA	14 (4.7%)
α-L + CA	14 (4.7%)
β-L + CA	7 (2.4%)
All IgE CMP allergens	3 (1.0%)

**Table 3:** The prevalence of CMP-sensitizing allergens in adults among other food allergens present in the food sIgE blood test panel (n = 295). ^*^Significant at Bonferroni-adjusted *P*-value (0.05/29). CMP, cow's milk protein; sIgE, specific immunoglobulin E

Food sIgE allergen	*n* (%)	*P*-value
β-Lactoglobulin	20 (6.8%)	<0.001*
Milk	22 (7.5%)	<0.001*
Orange	22 (7.5%)	<0.001*
Onion	32 (10.9%)	<0.001*
Cod fish	34 (11.5%)	<0.001*
Strawberry	34 (11.6%)	<0.001*
Tomato	34 (11.6%)	<0.001*
Soybean	34 (11.6%)	<0.001*
Mango	38 (12.9%)	<0.001*
Kiwi	38 (12.9%)	<0.001*
Egg yolk	39 (13.2%)	<0.001*
Peanut	39 (13.2%)	<0.001*
α-Lactalbumin	40 (13.5%)	<0.001*
Casein	43 (14.6%)	<0.001*
Mussel	43 (14.6%)	<0.001*
Cacao	44 (14.9%)	<0.001*
Carrot	45 (15.3%)	<0.001*
Pecan nut	45 (15.3%)	<0.001*
Cheese mix	47 (16.0%)	<0.001*
Wheat flour	47 (16.0%)	<0.001*
Banana	47 (16.0%)	<0.001*
Egg white	49 (16.6%)	<0.001*
Walnut	50 (16.9%)	<0.001*
Shrimps	52 (17.6%)	<0.001*
Date	56 (19.0%)	<0.001*
Fig	60 ( 20.3%)	<0.001*
Cashew nut	72 (24.4%)	<0.001*
Honey	73 (24.7%)	<0.001*
Sesame seed	112 (38.0%)	<0.001*

Prevalence of IgG CMPA: Of 314 adults enrolled in this study, the food sIgG level was measured in 92 (29.3%) adults. Table 4 summarizes the prevalence of IgG CMPA in adults for cow milk, cow rennet cheese, and cow sour milk products. Cow milk was the most frequent sensitizing allergen reported in 54 (58.7%) subjects, followed by cow sour milk products in 41 (44.6%) subjects. Thirty (32.6%) adults were shown to be sensitized to all CMP allergens in the IgG panel. Data showed that more allergy cases were significantly reported against the CMP profile (cow milk, cow rennet cheese, and cow sour milk products) present in the sIgG food testing panel compared to other food allergens in the same panel (e.g., chicken, salmon, and buckwheat). These findings were statistically calculated using the chi-square goodness-of-fit test. Following this, a post hoc examination was conducted using partitioning with a Bonferroni-adjusted *P*-value (0.05/53). Table 5 shows the prevalence of IgG CMP allergens in adults among other food allergens present in the IgG blood test panel.

**Table 4:** The prevalence of IgG CMPA in adults for cow milk, cow rennet cheese, and cow sour milk products (n = 92). Missing values are not considered in calculating the percentages. CMPA, cow's milk protein allergy; CMP, cow's milk protein; IgE, immunoglobulin E

Cow milk proteins (CMPs)	IgG CMPA reported cases, *n* (%)
Cow milk	54 (58.7%)
Cow Rennet cheese	32 (34.8%)
Cow sour milk products	41 (44.6%)
Cow milk + cow sour products	39 (42.4%)
Cow milk + cow Rennet cheese	32 (34.8%)
All CMP allergens	30 (32.6%)

**Table 5:** The prevalence of CMP-sensitizing allergens in adults among other food allergens present in the food sIgG blood test panel (n = 92). ^*^Significant at the Bonferroni-adjusted *P*-value (0.05/53). CMP, cow's milk protein; sIgE, specific immunoglobulin E

Food sIgG allergen	*n* (%)	*P*-value
Barley	74 (80.4%)	<0.001*
Wheat	70 (76.1%)	<0.001*
Gluten	67 (72.8%)	<0.001*
Oat	67 (72.8%)	<0.001*
Rye	67 (72.8%)	<0.001*
Spelt	63 (68.5%)	<0.001*
Cow milk	54 (58.7%)	<0.001*
Chicken egg white and yolk	43 (46.7%)	<0.001*
Cow sour milk products	41 (44.6%)	<0.001*
Goat milk and cheese	34 (37.0%)	<0.001*
Cow rennet cheese	32 (34.8%)	<0.001*
Sheep milk and cheese	31 (33.7%)	<0.001*
Green pea	25 (27.2%)	<0.001*
Yeast	24 (26.1%)	<0.001*
Peanut	21 (22.8%)	<0.001*
Soybean	19 (20.7%)	<0.001*
Honey mixture	16 (17.4%)	<0.001*
Pistachio	15 (16.3%)	<0.001*
Broccoli	14 (15.2%)	<0.001*
Pineapple	14 (15.2%)	<0.001*
Carrots	13 (14.1%)	<0.001*
Cherry	13 (14.1%)	<0.001*
Maize sweet corn	13 (14.1%)	<0.001*
Sunflower seeds	13 (14.1%)	<0.001*
Cocoa bean	12 (13.0%)	<0.001*
Beet root	11 (12.0%)	<0.001*
Coffee	10 (10.9%)	<0.001*
Cashew kernels	9 (9.8%)	<0.001*
Sesame	9 (9.8%)	<0.001*
Apple	8 (8.7%)	<0.001*
Aubergine	8 (8.7%)	<0.001*
Banana	8 (8.7%)	<0.001*
Cucumber	8 (8.7%)	<0.001*
Hazelnut	8 (8.7%)	<0.001*
Almond	7 (7.6%)	<0.001*
Fig	7 (7.6%)	<0.001*
Kiwi	7 (7.6%)	<0.001*
Strawberry	7 (7.6%)	<0.001*
Buckwheat	6 (6.5%)	0.0011
Onion	6 (6.5%)	0.0011
Orange	6 (6.5%)	0.0011
Olive	5 (5.4%)	0.0126
Salmon	5 (5.4%)	0.0126
White beans	5 (5.4%)	0.0126
Lemon	4 (4.3%)	0.0837
Mussel	4 (4.3%)	0.0837
Red beans	4 (4.3%)	0.0837
Watermelon	4 (4.3%)	0.0837
Apricot	3 (3.3%)	0.3348
Date	2 (2.2%)	0.8422
Mango	2 (2.2%)	0.8422
Avocado	1 (1.1%)	0.5712
Chicken	1 (1.1%)	0.5712

Determination of Total Serum IgE and Total Serum IgG Levels in the Adult Population With CMPA

Percentage of total IgE and total IgG: Out of the 224 adult subjects who had total serum IgE levels measured, 74 had both CMPA and total IgE levels detected in their blood. Results showed that 24 (32.4% of reported cases) subjects had a normal level of total IgE <100 IU/mL. The remaining 50 (67.5%) measurements indicated a high total IgE level. According to the total IgG results, 36 subjects had both CMPA and blood test results for total IgG out of the 116 subjects measured for total IgG. A total of 34 (94.4% of reported cases) subjects demonstrated a normal level of total IgG < 1,600 IU/mL, while 2 (5.5%) subjects showed a high level of total IgG. The results of total IgE and total IgG are shown in Table 6.

**Table 6:** Percentage of measured total serum IgE and total serum IgG levels in adult population with CMPA. CMPA, cow's milk protein allergy; IgE, immunoglobulin E; IgG, immunoglobulin G

Total IgE (*n* = 74)			
Normal IgE		High IgE	
n	%	n	%
24	32.4%	50	67.5%
Total IgG (*n* = 36)			
Normal IgG		High IgG	
n	%	n	%
34	94.4%	2	5.5%

The frequency of allergic patients about their serum 25OHD levels: In 109 adult participants where serum 25OHD levels were measured, the frequency of allergic patients with high total IgE levels was identified. As demonstrated in Table 7, subjects with vitamin D deficiency had the highest frequency of allergic patients (17, 74%) compared to those with insufficient vitamin D (43, 67.2%), and sufficient vitamin D levels (13, 59.1%).

**Table 7:** The frequency of allergic patients in relation to their serum 25-hydroxyvitamin D levels.

Participant’s group	Number of subjects with total IgE level in different serum 25-hydroxyvitamin D levels measured						
	Deficient (<10 ng/mL)		Insufficient (10-30 ng/mL)		Sufficient (30-100 ng/mL)		Total
Number of subjects with total IgE level measured in the adult group	23		64		22		109
Number of subjects with normal and high total IgE levels reported	Normal	High	Normal	High	Normal	High	-
	6	17	21	43	9	13	
Percentage of subjects with normal and high total IgE levels reported	26.0%	74.0%	32.8%	67.2%	41.0%	59.1%	

Association Between the Presence of CMPA With Gender, BMI, Vitamin D, and Presence of Inhalant Allergies

Gender, BMI, and vitamin D levels were found to have no significant relationship in the adult population (*P*-value > 0.05). A slight increase was found in females exhibiting confirmed CMPA in both IgE and IgG CMPA cases. Although the majority of CMPA cases demonstrated positive inhalant sanitization, there was no statistically significant relationship found between inhalant allergy and the presence of IgE or IgG CMPA cases. Tables 8-9 show the results of Pearson’s chi-square test to investigate the possible association between the presence of CMPA in both IgE and IgG forms with gender, BMI, vitamin D level, and the presence of inhalant allergies.

**Table 8:** Clinical characteristics of the adult population with confirmed IgE CMPA. BMI, body mass index; CMPA, cow's milk protein allergy; IgE, immunoglobulin E

Subjects with CMP IgE allergy										
Variables	Milk (*n* = 22)	No CMPA (*n *= 273)	α-Lactalbumin (*n *= 40)	No CMPA (*n *= 254)		β-Lactoglobulin (*n *= 20)	No CMPA (*n *= 271)	Casein (*n *= 43)	No CMPA (*n *= 250)	
	*n* (%)		*n* (%)			*n* (%)		*n* (%)		
Gender										
Male	8 (36.4%)	112 (41.0%)	18 (45%)	101 (39.8%)		11 (55%)	120 (44.3%)	19 (44.2%)	90 (36.0%)	
Female	14 (63.6%)	161 (59.0%)	22 (55%)	153 (60.2%)		9 (45%)	151 (55.7%)	24 (55.8%)	162 (64.8%)	
*P*-value	0.668		0.531			0.352		0.287		
Body weight status using BMI										
Underweight	1 (12.5%)	5 (6.0%)	1 (6.7%)	4 (55.5%)		1 (14.2%)	6 (6.5%)	1 (6.7%)	5 (6.0%)	
Normal	2 (25.0%)	18 (22%)	3 (20%)	17 (23.6%)		2 (28.6%)	20 (21.7%)	6 (40%)	16 (19.3%)	
Overweight	4 (50.0%)	27 (33%)	9 (60%)	22 (30.5%)		2 (28.6%)	33 (35.9%)	6 (40%)	29 (35.0%)	
Obese	1 (12.5%)	32 (39%)	2 (13.3%)	29 (40.3%)		2 (28.6%)	33 (35.9%)	2 (13.3%)	33 (39.7%)	
*P*-value	0.476		0.130			0.828		0.171		
Vitamin D level										
Deficiency	2 (33.3%)	24 (21.2%)	5 (33.3%)	21 (20.2%)		2 (22.3%)	24 (21.6%)	3 (17.6%)	23 (22.4%)	
Insufficiency	2 (33.3%)	67 (59.3%)	5 (33.3%)	64 (61.5%)		3 (33.3%)	67 (60.3%)	9 (52.9%)	61 (59.2%)	
Sufficiency	2 (33.3%)	22 (19.5%)	5 (33.3%)	19 (18.3%)		4 (44.4%)	20 (18.0%)	5 (29.4%)	19 (18.4%)	
*P*-value	0.452		0.116			0.139		0.570		
Presence of inhalant allergies										
Do not have	1 (4.5%)	22 (8.2%)	2 (5%)		21 (8.4%)	1 (5%)	23 (8.5%)	2 (4.7%)	20 (8.0%)	
Have	21 (95.5%)	247 (91.8%)	38 (95%)		229 (91.6%)	19 (95%)	248 (91.5%)	41 (95.3%)	230 (91.6%)	
*P*-value	0.544		0.460			0.584		0.441		

**Table 9:** Clinical characteristics of the adult population with confirmed IgG CMPA. CMPA, cow's milk protein allergy; IgG, immunoglobulin G; BMI, body mass index

Subjects with CMP IgG allergy										
Variables	Cow milk (*n *= 54)	No CMPA (*n *= 38)	Rennet cheese (*n *= 32)	No CMPA (*n *= 59)		Cow sour milk products (*n *= 41)			No CMPA (*n *= 51)	
	*n* (%)		*n* (%)			*n* (%)				
Gender										
Male	22 (40.7%)	13 (34.2%)	16 (50%)		20 (33.9%)	16 (39%)			19 (37.3%)	
Female	32 (59.3%)	25 (65.8%)	16 (50%)		40 (67.8%)	25 (61%)			32 (62.7%)	
*P*-value	0.525		0.119			0.862				
Body weight status using BMI										
Underweight	2 (10%)	1 (5.9%)	1 (10%)	2 (7.4%)		1 (6.7%)				2 (9.1%)
Normal	4 (20%)	3 (17.6%)	3 (30%)	4 (14.8%)		4 (26.7%)				3 (13.6%)
Overweight	8 (40%)	6 (35.3%)	5 (50%)	9 (33.3%)		6 (40%)				8 (36.4%)
Obese	6 (30%)	7 (41.2%)	1 (10%)	12 (44.4%)		4 (26.7%)				9 (40.9%)
*P*-value	0.897		0.267			0.703				
Vitamin D level										
Deficiency	3 (15%)	1 (9.1%)	0 (0%)	4 (19%)		1 (7.7%)		4 (21.1%)		
Insufficiency	14 (70%)	6 (54.5%)	9 (90%)	11 (52.4%)		11 (84.6%)		9 (47.4%)		
Sufficiency	3 (15%)	4 (36.4%)	1 (10%)	6 (28.6%)		1 (7.7%)		6 (31.6%)		
*P*-value	0.389		0.109			0.099				
Presence of inhalant allergies										
Do not have	7 (15.6%)	2 (6.9%)	5 (20%)	4 (8.2%)		5 (16.1%)	4 (9.3%)			
Have	38 (84.4%)	27 (93.1%)	20 (80%)	45 (91.8%)		26 (83.9%)	39 (90.7%)			
*P*-value	0.266		0.141			0.375				

Prevalence of the Most Associated Symptoms Reported Among Adult Patients With CMPA

Of the 314 adults enrolled in this study, 71 (22.6%) were both CMPA-positive and symptomatic. In total, 19 different symptoms were reported by adults with CMPA. Table 10 demonstrates symptoms reported by adults with positive CMPA. The most common symptoms among adults with CMPA were allergic rhinitis (AR, 24, 33.8%) and gastroesophageal reflux disease (GERD) (24, 33.8%). These were followed by eczema (22, 31%), dyspnea (21, 29.6%), urticaria (19, 26.8%), and constipation (12, 16.9%). AR, GERD, and eczema were significantly increased in the adult population with CMPA compared to skin rash, angioedema, and atopic dermatitis (AD). These findings were statistically calculated using the chi-square goodness-of-fit test followed by post hoc examination using partitioning with the Bonferroni-adjusted *P*-value (0.05/19).

**Table 10:** Clinical symptoms of adults confirmed with cow’s milk protein allergy (CMPA). ^*^Significant at the Bonferroni-adjusted *P*-value (0.05/19). IgE, immunoglobulin E

Symptoms	Symptoms reported by IgE CMPA, *n* (%) (*n* = 45)	Symptoms Reported by IgG CMPA, *n* (%) (*n* = 36)	Total symptomatic cases, *n* (%) (*n* = 71)	*P*-value
Allergic rhinitis (AR)	17 (37.8%)	10 (27.8%)	24 (33.8%)	<0.001*
Gastroesophageal reflux disease (GERD)	15 (33.3%)	13 (36.1%)	24 (33.8%)	<0.001*
Eczema	16 (35.6%)	9 (25%)	22 (31%)	<0.001*
Dyspnea	11 (24.4%)	14 (38.9%)	21 (29.6%)	<0.001*
Urticaria	12 (26.7%)	9 (25%)	19 (26.8%)	<0.001*
Constipation	6 (13.3%)	8 (22.2%)	12 (16.9%)	<0.001*
Asthma	7 (15.6%)	3 (8.3%)	9 (12.7%)	<0.001*
Sinusitis	8 (17.8%)	5 (13.9%)	9 (12.7%)	<0.001*
Diarrhea	3 (6.7%)	7 (19.4%)	8 (11.3%)	<0.001*
Conjunctivitis	4 (8.9%)	2 (5.6%)	6 (8.5%)	<0.001*
Skin rash	2 (4.4%)	2 (5.6%)	4 (5.6%)	0.053
Angioedema	1 (2.2%)	3 (8.3%)	3 (4.2%)	0.116
Atopic dermatitis (AD)	2 (4.4%)	0 (0%)	2 (2.8%)	0.127
Pruritus	2 (4.4%)	0 (0%)	2 (2.8%)	0.127
Irritable bowel syndrome (IBS)	0 (0%)	2 (5.6%)	2 (2.8%)	0.127
Rhino conjunctivitis	1 (2.2%)	1 (2.8%)	1 (1.4%)	0.070
Migraine	1 (2.2%)	0 (0%)	1 (1.4%)	0.070
Dandruff	1 (2.2%)	0 (0%)	1 (1.4%)	0.070
Abdominal pain	0 (0%)	1 (2.8%)	1 (1.4%)	0.070

Pediatric population

Descriptive Statistics

Sixty-two children (girls 34, 54.8%; boys 28, 45.2%) were included in this study. Table 11 summarizes the age, weight, and height of children included in this study. The BMI was only reported for 23 (37.1%) participants. The majority of the participants showed normal BMIs (16, 69.6%).

**Table 11:** Demographic characteristics of pediatric participants enrolled in the study. SD, standard deviation

Demographic characteristics	Mean ± SD
Age (years)	8.2 ± 4.4
Male, *n* (%)	28 (45.2%)
Female, *n* (%)	34 (54.8%)
Weight (kg)	34.3 ± 22.3
Height (cm)	128.9 ± 22
BMI (kg/m^2^)	18.8 ± 6
Underweight, *n* (%)	1 (4.3%)
Normal, *n* (%)	16 (69.6%)
Overweight, *n* (%)	2 (8.7%)
Obese, *n* (%)	4 (17.4%)

Prevalence of CMPA in Both IgE and IgG Forms

Prevalence of IgE CMPA: Food sIgE levels were measured in 58 (93.5%) children. Table 12 summarizes the prevalence of IgE CMPA in pediatrics for milk, α-lactalbumin, β-lactoglobulin, and casein allergen. The most common CMP IgE allergen observed was α-lactalbumin (20, 34.5%) followed by milk (11, 19%) and casein (10, 17.2%). All allergens were positive in three children. A higher number of allergy cases were significantly reported for all food allergens present in the sIgE panel, including the milk profile (casein, α-lactalbumin, milk, and β-lactoglobulin), among the adult population. These findings were statistically calculated using the chi-square goodness-of-fit test followed by post hoc examination using partitioning with the Bonferroni-adjusted P-value (0.05/29). Table 13 displays the prevalence of CMP-sensitizing allergens in the pediatric population, among other food allergens present in the sIgE blood test panel.

**Table 12:** The prevalence of sIgE CMPA in the pediatric population for milk α-L, β-L, and CA allergens (n = 58). ^*^Missing values are not considered in calculating the percentages. CA, casein; CMPA, cow's milk protein allergy; sIgE, specific immunoglobulin E

CMPs	IgE CMPA reported cases, *n* (%)
Milk	11 (19.0%)
α-Lactalbumin (α-L)	20 (34.5%)
β-Lactoglobulin (β-L)	9 (15.5%)
Casein	10 (17.2%)
Milk + α-L	7 (12.1%)
Milk + β-L	4 (6.9%)
α-L + β-L	7 (12.1%)
Milk + CA	5 (8.6%)
α-L + CA	5 (8.6%)
β-L + CA	4 (6.9%)
All IgE CMP allergens	3 (5.2%)

**Table 13:** The prevalence of CMP-sensitizing allergens in the pediatric population among other food allergens present in the food sIgE blood test panel (n = 58). ^*^Significant at the Bonferroni-adjusted *P*-value (0.05/29). sIgE, specific immunoglobulin E; CMP, cow's milk protein

Food sIgE allergen	*n* (%)	*P*-value
Cashew nut	36 (62.0%)	<0.001*
Egg white	26 (44.8%)	<0.001*
α-Lactalbumin	20 (34.5%)	<0.001*
Honey	20 (34.5%)	<0.001*
Wheat flour	16 (27.6%)	<0.001*
Carrot	16 (27.6%)	<0.001*
Peanut	16 (27.6%)	<0.001*
Cod fish	15 (25.9%)	<0.001*
Cacao	15 (25.9%)	<0.001*
Mussel	14 (24.1%)	<0.001*
Banana	14 (24.1%)	<0.001*
Egg yolk	13 (22.4%)	<0.001*
Fig	13 (22.4%)	<0.001*
Kiwi	13 (22.4%)	<0.001*
Cheese mix	12 (20.7%)	<0.001*
Shrimps	12 (20.7%)	<0.001*
Date	12 (20.7%)	<0.001*
Milk	11 (19.0%)	<0.001*
Sesame seed	11 (19.0%)	<0.001*
Casein	10 (17.2%)	<0.001*
β-Lactoglobulin	9 (15.5%)	<0.001*
Orange	8 (13.8%)	<0.001*
Pecan nut	8 (13.8%)	<0.001*
Walnut	8 (13.8%)	<0.001*
Strawberry	7 (12.0%)	<0.001*
Soybean	7 (12.0%)	<0.001*
Onion	6 (10.3%)	0.004
Mango	3 (5.2%)	0.472
Tomato	3 (5.2%)	0.472

Prevalence of IgG CMPA: Of the 62 children enrolled in this study, 20 (32%) were measured for food sIgG for cow milk, cow rennet cheese, and cow sour milk products. Table 14 summarizes the prevalence of CMPA in the pediatric population for cow milk, cow rennet cheese, and cow sour milk products. Cow milk was the most common sensitizing allergen (20, 100%) followed by cow sour milk products (14, 70%). Ten children showed to be positive for all CMPs IgG allergens. More allergy cases were significantly reported against the CMP profile (cow milk, cow rennet cheese, and cow sour milk products) present in the sIgG testing panel. These findings were statistically calculated using the chi-square goodness-of-fit test followed by post hoc examination using partitioning, with a Bonferroni-adjusted *P*-value (0.05/45). Table 15 shows the prevalence of CMP-sensitizing allergens in the pediatric population among other food allergens present in the food sIgG blood test panel.

**Table 14:** The prevalence of IgG CMPA in the pediatric population for cow milk, cow rennet cheese, and cow sour milk products (n = 20). Missing values are not considered in calculating the percentages. CMPA, cow's milk protein allergy; IgG, immunoglobulin G; CMP, cow's milk protein

CMPs	IgG CMPA reported cases, *n* (%)
Cow milk	20 (100%)
Cow Rennet cheese	12 (60%)
Cow sour milk products	14 (70%)
Cow milk + cow sour products	14 (70%)
Cow milk + cow Rennet cheese	12 (60%)
All IgG CMP allergens	10 (50%)

**Table 15:** The prevalence of CMP-sensitizing allergens in the pediatric population among other food allergens present in the food sIgG blood test panel (n = 20). ^*^Significant at the Bonferroni-adjusted *P*-value (0.05/45). sIgG, specific immunoglobulin G; CMP, cow's milk protein

Food sIgG allergen	*n* (%)	*P*-value
Cow milk	20 (100.0%)	<0.001*
Gluten	17 (85.0%)	<0.001*
Spelt	17 (85.0%)	<0.001*
Wheat	17 (85.0%)	<0.001*
Sheep milk and cheese	15 (83.3%)	<0.001*
Cow sour milk products	14 (70.0%)	<0.001*
Oat	16 (80.0%)	<0.001*
Cow rennet cheese	12 (60.0%)	<0.001*
Chicken eggs	14 (70.0%)	<0.001*
Goat milk and cheese	14 (70.0%)	<0.001*
Barley	12 (60.0%)	<0.001*
Hazelnut	10 (50.0%)	<0.001*
Pistachio	10 (50.0%)	<0.001*
Soybean	10 (50.0%)	<0.001*
Carrots	8 (40.0%)	<0.001*
Cashew	8 (40.0%)	<0.001*
Cucumber	7 (35.0%)	<0.001*
Honey	7 (35.0%)	<0.001*
Orange	7 (35.0%)	<0.001*
Almond	6 (30.0%)	<0.001*
Aubergine	6 (30.0%)	<0.001*
Cherry	6 (30.0%)	<0.001*
Peanut	6 (30.0%)	<0.001*
Apricot	5 (25.0%)	<0.001*
Sesame	5 (25.0%)	<0.001*
Watermelon	5 (25.0%)	<0.001*
Yeast	5 (25.0%)	<0.001*
Apple	4 (20.0%)	<0.001*
Beetroot	4 (20.0%)	<0.001*
Maize	4 (20.0%)	<0.001*
Mustard	4 (20.0%)	<0.001*
Potato	4 (20.0%)	<0.001*
Strawberry	4 (20.0%)	<0.001*
Banana	3 (15.0%)	<0.001*
Broccoli	3 (15.0%)	<0.001*
Kiwi	3 (15.0%)	<0.001*
Olives	3 (15.0%)	<0.001*
Onion	3 (15.0%)	<0.001*
Tomato	3 (15.0%)	<0.001*
Walnut	3 (15.0%)	<0.001*
Buck wheat	2 (10.0%)	0.019
Chicken	2 (10.0%)	0.019
Lemon	2 (10.0%)	0.019
Pineapple	2 (10.0%)	0.019
Salmon	2 (10.0%)	0.019

Determination of Total IgE and Total IgG Levels in the Pediatric Population With CMPA

Thirty-four pediatric subjects had total IgE levels measured. Twenty-four were both CMPA positive and had total IgE levels detected in their blood. Results showed that 10 (41.6% of reported cases) subjects had a normal level of total IgE <100 IU/mL. The remaining 58.3% who were measured showed a high total IgE level. According to total IgG results, 13 subjects were both CMPA positive and had blood test results for total IgG, 11 (84.6% of reported cases) subjects demonstrated normal levels of total IgG <1,560 IU/mL, while 2 (15.3%) subjects showed a high level of total IgG. The results of total IgE and total IgG are shown in Table 16.

**Table 16:** Percentage of total IgE and total IgG levels measured in the pediatric population with CMPA. CMPA, cow's milk protein allergy; IgE, immunoglobulin E; IgG, immunoglobulin G

Total IgE (*n *= 24)			
Normal IgE		High IgE	
n	%	n	%
10	41.6%	14	58.3%
Total IgG (*n *= 13)			
Normal IgG		High IgG	
n	%	n	%
11	84.6%	2	15.3%

Association Between the Presence of CMPA With Gender, Age, BMI, Vitamin D, Inhalant Allergies, and Other Parental and Environmental Characteristics

IgE CMPA: Among the 62 pediatric subjects included in the study, no significant association was observed between IgE CMPA cases and gender, BMI, and vitamin D levels. The presence of CMPA against β-lactoglobulin was significantly higher in children aged zero to five years (*P* = 0.005, *P* < 0.05) compared to those over five years old. The association was measured using Cramer's V calculation, revealing an effect size of 0.039, indicating a medium association. A significant association was not observed between CMPA and the presence of IgE inhalant allergies in the pediatric population, although more than 60% of children in each confirmed group of CMPA reported positive reactions to one or more IgE inhalant allergies. The child’s feeding pattern and type of delivery were not significantly associated with the presence of CMPA cases in affected children. Children who had contact with pets in their homes appeared to have a marginally significant reduced rate of CMPA cases against α-lactalbumin (*P* = 0.054). Table 17 summarizes the results of Pearson's chi-square test to investigate the possible association between the presence of CMPA in both IgE forms with gender, BMI, vitamin D, and inhalant allergies, in addition to other collected demographic characteristics.

**Table 17:** Clinical characteristics of the pediatric population with confirmed IgE CMPA. ^*^Significant at *P* < 0.01. ^**^Significant at *P* < 0.05. CMPA, cow's milk protein allergy; IgE, immunoglobulin E

Subjects with IgE CMP allergy											
Variables	Milk (*n* = 11)			No CMPA (*n *= 47)	α-Lactalbumin (*n *= 20)	No CMPA (*n *= 37)		β-Lactoglobulin (*n *= 9)	No CMPA (*n *= 49)	Casein (*n *= 10)	No CMPA (*n *= 48)
	*n* (%)				*n* (%)			*n* (%)		*n* (%)	
Gender											
Male	7 (63.6%)			19 (40.4%)	8 (40.0%)	17 (46.0%)		4 (44.4%)	22 (44.9%)	5 (50.0%)	21 (43.8%)
Female	4 (36.4%)			28 (59.6%)	12 (60.0%)	20 (54.0%)		5 (55.6%)	27 (55.1%)	5 (50.0%)	27 (56.3%)
*P*-value	0.163				0.665			0.979		0.718	
Age											
0-5 years	5 (45.5%)			15 (31.9%)	9 (45.0%)	10 (27.0%)		7 (77.8%)	13 (26.5%)	5 (50.0%)	15 (31.3%)
Over 5 years	6 (54.5%)			32 (68.1%)	11 (55.0%)	27 (73.0%)		2 (22.2%)	36 (73.5%)	5 (50.0%)	33 (68.8%)
*P*-value	0.395				0.169			0.005*		0.256	
Body weight status using BMI											
Underweight	1 (20.0%)			5 (6.0%)	1 (6.7%)	4 (55.5%)		1 (14.2%)	6 (6.5%)	1 (6.7%)	5 (6.0%)
Normal	4 (80.0%)			17(22%)	3 (20%)	15 (23.6%)		2 (28.6%)	15 (21.7%)	6 (40%)	15 (19.3%)
Overweight/obese	1 (20.0%)			25 (33%)	9 (60%)	19 (30.5%)		2 (28.6%)	28 (35.9%)	6 (40%)	28 (35.0%)
*P*-value	0.238				0.479			0.749		0.603	
Vitamin D level											
Deficiency	0 (33.3%)			5 (33.3%)	1 (11.1%)	4 (30.8%)		0 (03%)	5 (21.6%)	1 (20%)	4 (23.5%)
Insufficiency	6 (85.7%)			9 (60.0%)	7 (77.8%)	8 (61.5%)		5 (83.3%)	10 (60.3%)	3 (60%)	12 (70.6%)
Sufficiency	1 (14.3%)			1 (6.7%)	1 (11.1%)	1 (7.7%)		1 (16.7%)	1 (18.0%)	1 (20%)	1 (5.9%)
*P*-value	0.212				0.554			0.262		0.627	
Presence of inhalant allergies											
Do not have	2 (18.1%)		3 (6.8%)		1 (5.0%)		3 (8.3%)	0 (0%)	3 (7.0%)	1 (10.0%)	3 (6.5%)
Have	9 (81.8%)		41 (93.2%)		19 (95.0%)		33 (95.0%)	9 (100%)	43 (93.5%)	9 (90.0%)	43 (93.5%)
*P*-value	0.241				0.642			0.430		0.698	
Feeding pattern											
Breast milk	4 (40.0%)	8 (25.8%)			6 (33.3%)		6 (26.0%)	3 (33.3%)	9 (28.1%)	3 (50%)	9 (25.7%)
Mixed feeding	6 (60%)	23 (74.2%)			12 (66.6%)		17 (74.0%)	6 (66.6%)	23 (71.8%)	3 (50%)	26 (74.3%)
*P*-value	0.391				0.612			0.761		0.227	
Type of delivery											
Normal	7 (70.0%)	21 (77.8%)			13 (72.2%)		15 (79.0%)	8 (88.9%)	20 (71.4%)	4 (66.7%)	24 (77.4%)
Cesarean	3 (30.0%)	6 (22.2%)			5 (27.8%)		4 (21.0%)	1 (11.1%)	8 (28.6%)	2 (33.3%)	7 (22.6%)
*P*-value	0.624				0.633			0.288		0.574	
Presence of pets											
No	8 (80.0%)	21 (72.4%)			16 (89.0%)		13 (62.0%)	7 (77.8%)	22 (73.3%)	5 (83.35)	24 (72.7%)
Yes	2 (20.0%)	8 (27.6%)			2 (11.1%)		8 (38.0%)	2 (22.2%)	8 (26.7%)	1 (16.7%	9 (27.2%)
*P*-value	0.636				0.054**			0.789		0.584	
Family history											
No	5 (50.0%)	15 (44.1%)			7 (66.7%)		8 (30.8%)	5 (55.6%)	15 (42.8%)	3 (42.9%)	17 (46.0%)
Yes	5 (50.0%)	19 (55.9%)			6 (33.3%)		18 (69.2%)	4 (44.4%)	20 (57.1%)	4 (57.1%)	20 (54.0%)
*P*-value	0.743				0.162			0.495		0.880	

IgG CMPA: According to IgG CMPA, the data of 20 out of 62 children for food sIgG panel were analyzed. The sensitization was 100% in the pediatric population against cow milk allergen present in the sIgG panel. The presence of 100% sensitization for cow milk allergy in the whole collected 20 subjects prevented us from performing a suitable statistical investigation to assess the possible association between CMPA and non-CMPA in previously mentioned parameters.

Prevalence of Most Associated Symptoms Reported Among Pediatric Patients With CMPA

Of the 62 children enrolled in this study, 33 (53%) were both CMPA-positive and symptomatic. In total, 15 different symptoms were reported by children with CMPA. Table 18 shows symptoms observed in children with positive CMPA. The most common symptoms were eczema, reported in 13 (39.40%) cases, followed by AR reported in 8 (24.20%) cases, and AD reported in 7 (21.20%) cases. Data showed that eczema, AR, and AD were significantly higher in the pediatric population compared to GERD, constipation, and abdominal pain. These findings were statistically calculated using the chi-square goodness-of-fit test followed by post hoc examination using partitioning with the Bonferroni-adjusted *P*-value (0.05/15).

**Table 18:** Clinical symptoms of pediatrics confirmed with CMPA. ^*^Significant at the Bonferroni-adjusted *P*-value (0.05/15). CMPA, cow's milk protein allergy; IgE, immunoglobulin E; IgG, immunoglobulin G

Symptoms	Symptoms reported by IgE CMPA, *n* (%) (*n* = 24)	Symptoms reported by IgG CMPA, *n* (%) (*n* = 14)	Total symptomatic cases, *n* (%) (*n* = 33)	*P*-value
Eczema	9 (37.5%)	4 (28.6%)	13 (39.4%)	<0.001*
Allergic rhinitis (AR)	7 (29.2%)	1 (7.1%)	8 (24.2%)	<0.001*
Atopic dermatitis (AD)	6 (25.0%)	3 (21.4%)	7 (21.2%)	<0.001*
Asthma	3 (13.0%)	1 (7.1%)	3 (9.4%)	<0.001*
Urticaria	3 (12.5%)	1 (7.1%)	3 (9.1%)	<0.001*
Vomiting	1 (4.2%)	3 (21.4%)	3 (9.1%)	<0.001*
Coughing	2 (8.3%)	1 (7.1%)	3 (9.1%)	<0.001*
Gastroesophageal reflux disease (GERD)	0 (0%)	2 (14.3%)	2 (6.1%)	0.007
Rhino conjunctivitis	2 (8.3%)	0 (0%)	2 (6.1%)	0.007
Skin rash	1 (4.2%)	1 (7.1%)	2 (6.1%)	0.007
Constipation	1 (4.2%)	0 (0%)	1 (3.0%)	0.006
Dyspnea	1 (4.2%)	0 (0%)	1 (3.0%)	0.006
Abdominal pain	1 (4.2%)	0 (0%)	1 (3.0%)	0.006
Eosinophilic esophagitis (EOE)	1 (4.2%)	1 (7.1%)	1 (3.0%)	0.006
Angioedema	1 (4.2%)	1 (7.1%)	1 (3.0%)	0.006

## Discussion

The health issue surrounding CMPA has become more prevalent in recent times, particularly in infants and children [5]. It is impossible to completely avoid exposure to CMP through food, inhalation, or direct contact. As a result, this condition has a negative impact on the quality of life of those who are affected, particularly children. Regarding CMP sensitization in the Saudi Arabian population, various studies have been conducted in the city of Riyadh during 1998 and 2016, respectively. The results indicated that CM was among the common food allergens in Saudi Arabia that cause both IgE and IgG reactions at prevalence rates of 12.9% and 56.3% among adults, respectively [22,23]. The majority of these food-related studies focused on determining the prevalence of most allergy-causing food allergens, including CM; this was preferred over investigating the prevalence of CMPA using CMP subclasses such as α-lactalbumin, β-lactoglobulin, and casein among different genders and age groups [24,25].

Our study was performed to assess the prevalence of CMPA among both adults and the pediatric population. We compared the prevalence of CMP allergens to the prevalence of other food allergens present in the food-specific IgE and IgG blood test panels. To gain additional insight into CMPA, we investigated the prevalence of CMP subclasses. The results showed prevalence rates of 7.5%, 13.5%, 6.8%, and 14.6% for sIgE against cow milk, α-lactalbumin, β-lactoglobulin, and casein, respectively. Casein was reported as the most predominant protein among adults with CMPA, producing higher levels of sIgE followed by α-lactalbumin. These findings were consistent with the findings of other food allergy studies [7,26]. This increase in casein may be explained by the fact that casein protein makes up the majority of the content of cow milk (80%) in addition to the increased consumption of casein protein in various items [27,28]. These items include coffee, extenders to nondairy products, and protein supplementation products consumed by athletes; usage rates of these products have reached approximately 61.6 % by Saudi men trained in fitness centers [29]. However, our results exhibited that CMP sensitization in adults is still a rare case when compared to sesame seed, honey, and cashew nut sensitization, showing 38%, 24.7%, and 24.4% sensitization rates, respectively.

Among the pediatric population, α-lactalbumin was the most reported sensitized CMP of the IgE type; prevalence rates for this CMP were 34.5% followed by 19% for cow milk. This finding is consistent with a Taiwan study that measured CMPs circulating sIgE in 190 children; it was found that α-lactalbumin had the greatest percentage followed by β-lactoglobulin in the research group [30]. Moreover, a Saudi study performed in the city of Jeddah also demonstrated an increase in α-lactalbumin as the main inducer for sensitization in patients with CMPA [31]. On the other hand, a study in the United States revealed that casein protein was the most CMP-eliciting sensitization reaction in 140 patients with CMPA aged six months to 22 years [7]. α-Lactalbumin is utilized in several commercially available infant formulae due to its various features; it contains necessary growth elements similar to those found in human breast milk, and it also possesses other physiological features, such as heat stability, which make it appropriate for use in many food, beverage and therapeutic products [32].

The reported sensitization cases of CMP subclasses are greater in number than the cases reported against whole milk. A possible explanation for this might be that cow’s milk is commonly available only after going through a technological procedure that usually involves heating. These procedures typically require high temperatures of up to 100 °C. In certain cases, temperatures above this degree are used to eradicate pathogenic bacteria or to produce milk in powdered forms, such as infant-formulated milk. The allergenicity and antigenicity of CM were shown to be affected by such high levels of heating and heating-activated changes in particular epitopes causing them to lose their ability to bind to sIgE. However, some linear epitopes stay unchanged and keep their allergenic potential even after heating [27]. On the other hand, some studies have documented the formation of new protein polymers capable of binding to sIgE after exposure to heating processes; this was seen in some of the CMP subclasses [33]. Nevertheless, differences in the prevalence of CMPs eliciting allergic reactions in patients with CMPA could be related to differences in race or geographic location. As seen in the Taiwan and Tayeb studies, as well as our findings, the Asian population reported a rise in α-lactalbumin. While Western countries reported a different increase in another CMP subclass (casein) [30,31]. However, unlike the results within the adult population, the pediatric population demonstrated that the CMP subclass in our study (α-lactalbumin) was among the top three food allergens producing allergic reactions. This confirms the fact that CMPA is more prevalent in the pediatric population, particularly in children under the age of five [2].

Regarding IgG sensitization results in response to the cow milk allergen, cow rennet cheese, and cow sour milk products; we found that cow milk had the highest IgG level with a prevalence rate of up to 100% in pediatric participants and 58.7% in adult participants. The high prevalence of CM IgG was also reported in the study by AlKhateeb [34]. This study revealed that CMP and CM derivative foods caused a stronger reaction to sIgG, particularly in children under the age of five [34]. The most likely cause of this finding could be due to consuming cow milk and its products regularly. As the actual role of sIgG in food allergies is unknown, several studies have concluded that having a high level of IgG is linked to increased permeability of gut mucosal cells. This allows food molecules to flow into the bloodstream, causing the subsequent development of a high level of IgG. However, high levels of circulating IgG in children under the age of five years may be attributed to the immaturity of intestinal mucosal cells in that age group, and it may generate a different immune response as the child grows older [10,35,36]. Studies have shown that the presence of a high level of IgG was reported more among CMPA-tolerant children than those who have a CMPA; this supports the opinion that IgG could be a tool to assess tolerance more than its diagnostic value [37]. On the other hand, some studies contradict our findings, reporting different types of food allergens as the main triggers for food allergy. In East Asia, chickpeas and shellfish were identified as the main food triggers while buckwheat was the main trigger in India, Hong Kong, and Korea [38]. This appears to confirm the fact that both geographical location and feeding patterns play a role in the wide range of prevalence among these studies.

In this study, the total serum IgE level was measured in patients with CMPA. The total serum IgE level was found to be elevated in 67.5% of the adult population and 58.3% of the pediatric population. These findings support prior studies that identified a relationship between a high level of total serum IgE in the blood and the existence of a variety of allergy disorders [39]. The association between the total IgE and vitamin D concentration was examined in the present study among 109 adult participants. According to the findings, participants with vitamin D deficiency had the highest prevalence of high total serum IgE levels (74%) of the participants compared to those with insufficiency (67.2%) and sufficiency levels (59.1%). This finding comes along with the previous scientific evidence showing that an adequate concentration of vitamin D in the blood can help suppress B-cell hyperactivity and lower the number of released Igs that can trigger an allergic reaction (i.e., IgE and cytokines) [40,41]. These findings were also observed in Abbas et al.’s recent study performed in the city of Jeddah; it was shown that the majority of allergic patients with an elevated serum level of IgE appeared to have deficient vitamin D concentrations in their blood [42].

In both the adult and pediatric populations, the association between IgE and IgG CMPA showed no significant differences in gender, BMI, and the level of vitamin D. However, the link between BMI and the presence of allergic disease was previously investigated in literature; changes in the levels of adipokines (adiponectin, leptin). Cytokines (IL6) in adipose tissue were reported in overweight and obese subjects, leading to impaired immune tolerance against allergic diseases [43]. Additionally, a Swedish study linked the presence of asthma with increasing BMI in their study population [44]. In examining this relationship, the inconsistencies between the results of our study and those of other studies may be due to differences in the types of allergic diseases being investigated. For instance, along with other studies about atopic dermatitis in children, the association between asthma and obesity among children was confirmed in an Australian study. However, most of the adult population in our study was classified as overweight or obese, which could be explained by the fact that most physicians evaluate the BMI for patients who appear to have weight problems. In addition to that, no studies have been conducted about the link between BMI and CMPA, limiting the possibility of comparison [45,46]. Despite this fact, our data revealed a significant increase in IgE CMPA cases in the pediatric population, particularly in the age group of children under years old (*P* = 0.003). This is consistent with findings in existing literature, suggesting that CMPA is a disorder of infancy and early childhood [9, 47]. The varying reactivity of CMPA with age may be explained by the immaturity of mucosal cells in the intestines of children in that age group. Additionally, different reactions may develop as the child grows, and an increase occurs in the number of immune cells, such as T-cells, which have a significant inhibitory influence on IgE production [9,35].

The relationship between the vitamin D level and the presence of CMPA was investigated regarding the adult population in the present study, and no significant association was observed (*P* > 0.05). This finding is in line with a 2006 United States study that utilized data from the National Health and Nutrition Survey; no link was found between low 25OHD and allergic sensitization, particularly in adults [48,49]. In contrast, another study conducted on a mice model demonstrated that vitamin D deficiency aggravated food allergy symptoms in those mice [50]. About the pediatric population, no significant association was observed between the presence of IgE CMPA and the level of vitamin D. A study conducted in a Chinese trial on vitamin D levels in children with CMPA demonstrated the vital role of vitamin D in eosinophilic migration, preventing blood eosinophilia symptoms associated with CMPA. The study stated that the decreased level of vitamin D observed in infants was due to the mother’s inadequate level of vitamin D during pregnancy [51]. In addition, another study was performed on 120 children below two years of age. Compared to the control group, a low level of vitamin D was found in CMP-allergic children who were exclusively breastfed [52]. However, the differences in the results between studies concerning this relationship may be due to a variety of factors that influence vitamin D levels, including race, age, genetic variation, and geographic factors [48-50]. Regarding the Saudi population, estimating the true association may be challenging due to factors such as differences in diet patterns within various Saudi Arabian cities and the wide variety in resulting ranges of serum vitamin D tests between laboratories caused by variations in analysis methods used.

In our study, results on the existence of inhalant CMPA sensitization within the study population did not show a significant association (*P* > 0.05), as indicated in the tables. However, a predominance of positive inhalant sensitization was observed for IgE and IgG cases among both adult and pediatric CMP-allergic participants. This increased number of documented aeroallergen cases in our study is accompanied by an increase in reported cases of AR symptoms as well; it appears that this is the most reported symptom in adults and the second most reported symptom in children. Alternatively, the cross-reactivity between aeroallergens and food allergens in IgE-sensitized patients with respiratory symptoms was documented in cases of pollen-food syndromes, including mite-shrimp and bird-egg syndromes [53]. Other opinions indicated that early sensitization to food in infancy could develop into inhalant allergies as the child grows older [54]. In addition, Jeddah’s year-round warm and humid temperature could potentially provide an ideal habitat for a high concentration of the dust mite allergen in homes, provoking respiratory allergies [42].

In this study, additional parental and environmental factors were analyzed within the pediatric population; these factors included the type of feeding infants received (breastmilk feeding or mixed feeding with formula containing CMP) and the method by which the infants were delivered. No significant association existed with the type of feeding that a child with IgE CMPA received. Similarly, no significant association was found between children who were born naturally and those who were born by cesarean section. Our findings contradict the findings from a 2018 study in Poland, which concluded that breastfeeding lowered the risk of CMPA in the study population. However, these findings were consistent with our findings in that the type of delivery showed no association with the presence of CMPA in the population studied [55]. By comparison, a Chinese trial found that shortening the period of infant breastfeeding, using formulated milk and cesarean delivery are risk factors for developing CMPA in children [47]. The real causes of such findings are not well defined in the literature. However, scientific opinion has indicated that the altered composition of the gut microbiota, particularly in the early years of life, influences the development of food allergies like CMPA, and this composition is influenced by factors like diet, lifestyle, delivery method, and feeding habits [56,57]. However, some scientists suggest that the use of probiotics and prebiotics may be considered a promising therapeutic protocol for the resolution of CMPA, emphasizing the demand for large-scale studies to confirm these findings [17,18].

The existence of allergies in the CMPA child’s family, including that of their parents and/or siblings, was studied in the present study. The findings revealed no association between IgE CMPA and family history. This relationship was approved in the study performed in Poland in which the effect of parental and environmental factors was evaluated regarding the CMP sensitivities of 138 infants who were confirmed to be affected. The prevalence rate was three times higher in the presence of a positive family history of allergy in the parents and twice as high in that of one affected sibling [55]. Furthermore, the Chinese trial also reported a strong association between CMPA in children and the presence of food allergy in one or both parents [47]. Additionally, the environmental aspect of having a pet in the child's home was also examined. A marginally significant decrease in CMPA cases was found in children in contact with pets (*P* = 0.054). As explained by the hygienic theory; keeping a pet in the home has been shown to reduce the incidence of CMPA based on the results of the study by Sardecka et al. [55]. However, the low number of collected data on families of CMPA children besides a low number of children who own pets in our study population limited the ability to draw a significant conclusion regarding these factors. This may be due to raising a pet in the home is still uncommon in Saudi Arabian society.

The IgE-mediated form of CMPA was found to be responsible for most of the symptoms seen in our study population. As seen in the results showed that AR, GERD, and eczema were the most frequent symptoms reported among adults, accounting for 33.8%, 33.8%, and 31%, respectively, of all CMPA cases (*n* = 71). Moreover, these symptoms also showed a significantly increased rate compared to irritable bowel syndrome, abdominal pain, and angioedema (*P* < 0.001). These symptoms have been previously identified in the literature among CMPA adults, with variation in reported prevalence [10,58,59]. From the total number of cases among symptomatic CMPA children (*n* = 33), eczema was the most reported manifestation (39.40%), followed by AR (24.20%) and AD (21.20%), with a significant increase (*P* < 0.001) reported in these manifestations. According to prior studies, 60% to 70% of CMPA symptoms in children appear as eczema, urticaria, and rashes on the skin; followed by gastrointestinal symptoms in 50% to 60% of children and respiratory symptoms in 20% to 30% of children [5,6,13]. Among Chinese infants, eczema was shown to be the most reported symptom (52.9%) [47]. In our study population, AR was found to be one of the most common manifestations. In numerous trials and reviews, AR is described as the most common respiratory symptom in patients with CMPA [6]. The rise in AR symptoms among patients with CMPA was also seen in the study by Tayeb et al. carried out in Jeddah; among 83 participants with confirmed CMPA cases, a remarkably high number of AR cases was found [29]. As documented in both the adult and pediatric populations of our study, increased positive inhalant results may explain the rise in AR cases reported. In addition, Jeddah’s year-round warm and humid temperature may provide an ideal habitat for a high concentration of the dust mite allergen in homes, provoking respiratory allergies. However, the symptoms in adults with CMPA tend to be more severe and persist for longer periods than those in children. These cases are also associated with increasing reports of anaphylactic shock in some studies [58].

Our study had several limitations. First, there was a small amount of collected CMPA patient data, especially among the pediatric population. This limited the statistically significant level of the results. Additionally, the blood concentration of sIgE and sIgG was used to diagnose CMPA. However, the Oral Food Challenge Test (OFCT) that accurately diagnoses this type of food allergy was not performed. This may lead to the over-diagnosis of CMPA in participants within the study population. Furthermore, this study was performed in the city of Jeddah and may not represent other regions of Saudi Arabia. Thus, large-scale studies and cohort studies with control groups are needed to assess more variables regarding CMPA in the Saudi population. Additional studies are also required to assess the usefulness of sIgG as a diagnostic tool in CMPA cases as well as the role of vitamin D in this allergic disorder. In addition, we aim to perform follow-up studies to design intervention diets. These diets may reduce symptoms by modulating the immune system and the gut microbiota. An example of the foods prescribed in these diets includes plant-based foods rich in polyphenols and naturally occurring prebiotics.

## Conclusions

In conclusion, casein and α-lactalbumin are the most common sensitizing allergens in IgE CMPs in adults and pediatrics, respectively. Cow milk was the most sensitizing allergen in IgG CMPs within the study population. Children under the age of five possess a higher rate of IgE CMPA cases, while children who own pets appear to have a lower rate of this reaction.

Further large-scale studies in different regions of Saudi Arabia are needed to assess the prevalence and risk factors associated with CMPA. We raised a recommendation for SFDA to impose regulations to protect individuals with CMPA allergies. This suggestion included ensuring appropriate and clear labeling of allergenic ingredients, such as milk and milk products, used in different commercially available food items.

## References

[1] Høst A (1994). Cow’s milk protein allergy and intolerance in infancy. Pediatr Allergy Immunol.

[2] Caffarelli C, Baldi F, Bendandi B, Calzone L, Marani M, Pasquinelli P (2010). Cow's milk protein allergy in children: a practical guide. Ital J Pediatr.

[3] (2023). Announcement Regarding Food Allergens Control.. https://old.sfda.gov.sa/en/food/circulations/Circulations/Food_Allergens_Control.pdf..

[4] Hochwallner H, Schulmeister U, Swoboda I, Spitzauer S, Valenta R (2014). Cow's milk allergy: from allergens to new forms of diagnosis, therapy and prevention. Methods.

[5] Host A, Halken S (2014). Cow's milk allergy: where have we come from and where are we going?. Endocr Metab Immune Disord Drug Targets.

[6] El-Agamy E (2007). The challenge of cow milk protein allergy. Small Rumin Res.

[7] Shek LP, Bardina L, Castro R, Sampson HA, Beyer K (2005). Humoral and cellular responses to cow milk proteins in patients with milk-induced IgE-mediated and non-IgE-mediated disorders. Allergy.

[8] Beyer K, Teuber SS (2005). Food allergy diagnostics: scientific and unproven procedures. Curr Opin Allergy Clin Immunol.

[9] Fiocchi A, Bognanni A, Brożek J, Ebisawa M, Schünemann H (2022). World Allergy Organization (WAO) diagnosis and rationale for action against cow's milk allergy (DRACMA) guidelines update - I - plan and definitions. World Allergy Organ J.

[10] Anthoni S, Savilahti E, Rautelin H, Kolho KL (2009). Milk protein IgG and IgA: the association with milk-induced gastrointestinal symptoms in adults. World J Gastroenterol.

[11] Schuyler AJ, Wilson JM, Tripathi A (2018). Specific IgG(4) antibodies to cow's milk proteins in pediatric patients with eosinophilic esophagitis. J Allergy Clin Immunol.

[12] Jenmalm MC, Björkstén B (19981). Exposure to cow’s milk during the first 3 months of life is associated with increased levels of IgG subclass antibodies to β-lactoglobulin to 8 years. J Allergy Clin Immunol.

[13] Lifschitz C, Szajewska H (2015). Cow's milk allergy: evidence-based diagnosis and management for the practitioner. Eur J Pediatr.

[14] Vandenplas Y, Al-Hussaini B, Al-Mannaei K (2019). Prevention of allergic sensitization and treatment of cow’s milk protein allergy in early life: the middle-east step-down consensus. Nutrients.

[15] Luyt D, Ball H, Makwana N, Green MR, Bravin K, Nasser SM, Clark AT (2014). BSACI guideline for the diagnosis and management of cow's milk allergy. Clin Exp Allergy.

[16] Sicherer SH, Sampson HA (2018). Food allergy: a review and update on epidemiology, pathogenesis, diagnosis, prevention, and management. J Allergy Clin Immunol.

[17] Qamer S, Deshmukh M, Patole S (2019). Probiotics for cow's milk protein allergy: a systematic review of randomized controlled trials. Eur J Pediatr.

[18] Osborn DA, Sinn JK (2007). Prebiotics in infants for prevention of allergic disease and food hypersensitivity. Cochrane Database Syst Rev.

[19] Kozłowska-Jalowska A, Horvath A, Vandenplas Y, Szajewska H (2021). Retrospective and prospective determination of the cow’s milk-related symptom score (Comiss™) values in symptomatic infants. Pediatr Gastroenterol Hepatol Nutr.

[20] Tayeb M, Alhussaini B, Qutub M (2020). The allergic diseases commonly associated with cow milk protein sensitization: a retrospective study (Jeddah-Saudi Arabia). Middle East J Fam Med.

[21] Mchd EJR (2011). Vitamin D and health: a historical overview. SA Orthop J.

[22] El-Rab MO (1998). Foods and food allergy: the prevalence of IgE antibodies specific for food allergens in Saudi patients. Saudi J Gastroenterol.

[23] Shakoor Z, AlFaifi A, AlAmro B, AlTawil LN, AlOhaly RY (2016). Prevalence of IgG-mediated food intolerance among patients with allergic symptoms. Ann Saudi Med.

[24] Aba-alkhail BA, El-Gamal FM (2000). Prevalence of food allergy in asthmatic patients. Saudi Med J.

[25] Sheikh F, Amin R, Rehan Khaliq AM, Al Otaibi T, Al Hashim S, Al Gazlan S (2015). First study of pattern of anaphylaxis in a large tertiary care hospital in Saudi Arabia. Asia Pac Allergy.

[26] Docena GH, Fernandez R, Chirdo FG, Fossati CA (1996). Identification of casein as the major allergenic and antigenic protein of cow's milk. Allergy.

[27] Restani P, Ballabio C, Di Lorenzo C, Tripodi S, Fiocchi A (2009). Molecular aspects of milk allergens and their role in clinical events. Anal Bioanal Chem.

[28] (2023). WHO/IUIS Allergen Nomenclature Home Page. http://www.allergen.org/..

[29] AlRuthia Y, Balkhi B, Alrasheed M, Altuwaijri A, Alarifi M, Alzahrani H, Mansy W (2018). Use of dietary and performance-enhancing supplements among male fitness center members in Riyadh: a cross-sectional study. PLoS One.

[30] Chen FM, Lee JH, Yang YH, Lin YT, Wang LC, Yu HH, Chiang BL (2014). Analysis of α-lactalbumin-, β-lactoglobulin-, and casein-specific IgE among children with atopic diseases in a tertiary medical center in Northern Taiwan. J Microbiol Immunol Infect.

[31] Tayeb MMS, Alhussaini B, Waked MS (2020). The allergic diseases commonly associated with cow milk protein sensitization: a retrospective study (Jeddah, Saudi Arabia). World Fam Med Journal/Middle East J Fam Med.

[32] Layman DK, Lönnerdal B, Fernstrom JD (2018). Applications for α-lactalbumin in human nutrition. Nutr Rev.

[33] Restani P, Ballabio C, Cattaneo A, Isoardi P, Terracciano L, Fiocchi A (2004). Characterization of bovine serum albumin epitopes and their role in allergic reactions. Allergy.

[34] Alkhateeb AF (2020). Foods causing highest IgG immune response in Saudi Arabia. Annu Res Rev Biol.

[35] Dannaeus A, Inganäs M (1981). A follow-up study of children with food allergy. Clinical course in relation to serum IgE- and IgG-antibody levels to milk, egg and fish. Clin Allergy.

[36] Antico A, Pagani M, Vescovi PP, Bonadonna P, Senna G (2011). Food-specific IgG4 lack diagnostic value in adult patients with chronic urticaria and other suspected allergy skin symptoms. Int Arch Allergy Immunol.

[37] Savilahti EM, Rantanen V, Lin JS (2010). Early recovery from cow's milk allergy is associated with decreasing IgE and increasing IgG4 binding to cow's milk epitopes. J Allergy Clin Immunol.

[38] Loh W, Tang ML (2018). The epidemiology of food allergy in the global context. Int J Environ Res Public Health.

[39] Satwani H, Rehman A, Ashraf S, Hassan A (2009). Is serum total IgE levels a good predictor of allergies in children?. J Pak Med Assoc.

[40] Pichler J, Gerstmayr M, Szépfalusi Z, Urbanek R, Peterlik M, Willheim M (2002). 1α,25(OH)2D3 inhibits not only Th1 but also Th2 differentiation in human cord blood T cells. Pediatr Res.

[41] Chen S, Sims GP, Chen XX, Gu YY, Chen S, Lipsky PE (2007). Modulatory effects of 1,25-dihydroxyvitamin D3 on human B cell differentiation. J Immunol.

[42] Abbas AA, Wajeeh J, Wajeeh H, Alghamdi A, Al Sharief S (2020). Vitamin D deficiency and its relation to allergic diseases: a cross sectional study among allergic patients from Jeddah City, Saudi Arabia. J Clin Immunol Microbiol.

[43] Hersoug LG, Linneberg A (2007). The link between the epidemics of obesity and allergic diseases: does obesity induce decreased immune tolerance?. Allergy.

[44] Bråbäck L, Hjern A, Rasmussen F (2005). Body mass index, asthma and allergic rhinoconjunctivitis in Swedish conscripts-a national cohort study over three decades. Respir Med.

[45] Tai A, Volkmer R, Burton A (2009). Association between asthma symptoms and obesity in preschool (4-5 year old) children. J Asthma.

[46] Silverberg JI (2011). Role of childhood obesity in atopic dermatitis. Expert Rev Dermatol.

[47] Yang M, Tan M, Wu J (2019). Prevalence, characteristics, and outcome of cow’s milk protein allergy in Chinese infants: a population-based survey. J Parenter Enteral Nutr.

[48] Lester MR (2011). Vitamin D levels and food and environmental allergies in the United States: results from the National Health and Nutrition Examination Survey 2005-2006. Pediatrics.

[49] Matsui T, Tanaka K, Yamashita H (2019). Food allergy is linked to season of birth, sun exposure, and vitamin D deficiency. Allergol Int.

[50] Matsui T, Yamashita H, Saneyasu KI, Tanaka H, Ito K, Inagaki N (2018). Vitamin D deficiency exacerbates sensitization and allergic diarrhea in a murine food allergy model. Allergol Int.

[51] Li J, Mei X, Cai X (2019). Association of blood eosinophilia and vitamin D insufficiency in young infants with cow milk allergy. Asia Pac J Clin Nutr.

[52] Silva CM, Silva SA, Antunes MM, Silva GA, Sarinho ES, Brandt KG (2017). Do infants with cow's milk protein allergy have inadequate levels of vitamin D?. J Pediatr (Rio J).

[53] Popescu FD (2015). Cross-reactivity between aeroallergens and food allergens. World J Methodol.

[54] Hattevig G, Kjellman B, Björkstén B (1993). Appearance of IgE antibodies to ingested and inhaled allergens during the first 12 years of life in atopic and non-atopic children. Pediatr Allergy Immunol.

[55] Sardecka I, Łoś-Rycharska E, Ludwig H, Gawryjołek J, Krogulska A (2018). Early risk factors for cow's milk allergy in children in the first year of life. Allergy Asthma Proc.

[56] Bunyavanich S, Shen N, Grishin A (2016). Early-life gut microbiome composition and milk allergy resolution. J Allergy Clin Immunol.

[57] Di Costanzo M, Carucci L, Berni Canani R, Biasucci G (2020). Gut microbiome modulation for preventing and treating pediatric food allergies. Int J Mol Sci.

[58] Lam HY, van Hoffen E, Michelsen A, Guikers K, van der Tas CH, Bruijnzeel-Koomen CA, Knulst AC (2008). Cow's milk allergy in adults is rare but severe: both casein and whey proteins are involved. Clin Exp Allergy.

[59] Stöger P, Wüthrich B (1993). Type I allergy to cow milk proteins in adults. A retrospective study of 34 adult milk- and cheese-allergic patients. Int Arch Allergy Immunol.

